# Myosin light chain kinase inhibitor ML7 improves vascular endothelial dysfunction via tight junction regulation in a rabbit model of atherosclerosis

**DOI:** 10.3892/mmr.2015.3973

**Published:** 2015-06-22

**Authors:** XIAOWEN CHENG, XIAOBIAN WANG, YUFENG WAN, QING ZHOU, HUAQING ZHU, YUAN WANG

**Affiliations:** 1 Department of Clinical Laboratory, The First Affiliated Hospital of Anhui Medical University, Hefei, Anhui 230032, P.R. China; 2 Laboratory of Molecular Biology and Department of Biochemistry, Anhui Medical University, Hefei, Anhui 230032, P.R. China; 3 Department of Otolaryngology, The Affiliated Chaohu Hospital of Anhui Medical University, Hefei, Anhui 230032, P.R. China

**Keywords:** atherosclerosis, myosin light chain kinase, vascular endothelial dysfunction, tight junction, ML7

## Abstract

Vascular endothelial dysfunction (VED) is an important factor in the initiation and development of atherosclerosis (AS). Previous studies have demonstrated that endothelial permeability is increased in diet-induced AS. However, the precise underlying mechanisms remain poorly understood. The present study aimed to analyze whether the myosin light chain kinase (MLCK) inhibitor ML7 is able to improve VED and AS by regulating the expression of the tight junction (TJ) proteins zona occludens (ZO)-1 and occludin via mechanisms involving MLCK and MLC phosphorylation in high-fat diet-fed rabbits. New Zealand white rabbits were randomly divided into three groups: Control group, AS group and ML7 group. The rabbits were fed a standard diet (control group), a high-fat diet (AS group) or a high-fat diet supplemented with 1 mg/kg/day ML7 (ML7 group). After 12 weeks, endothelium-dependent relaxation and endothelium-independent relaxation were measured using high-frequency ultrasound. Administration of a high-fat diet significantly increased the levels of serum lipids and inflammatory markers in the rabbits in the AS group, as compared with those in the rabbits in the control group. Furthermore, a high-fat diet contributed to the formation of a typical atherosclerotic plaque, as well as an increase in endothelial permeability and VED. These symptoms of AS were significantly improved following treatment with ML7, as demonstrated in the ML7 group. Hematoxylin & eosin and immunohistochemical staining indicated that ML7 was able to decrease the expression of MLCK and MLC phosphorylation in the arterial wall of rabbits fed a high-fat diet. A similar change was observed for the TJ proteins ZO-1 and occludin. In addition, western blot analysis demonstrated that ML7 increased the expression levels of occludin in the precipitate, but reduced its expression in the supernatant of lysed aortas. These results indicated that occludin, which is a dynamic protein at the TJ, is associated with remodeling from cell membrane to cytoplasm. The present study was the first, to the best of our knowledge, to indicate that ML7 may ameliorate VED and AS by regulating the TJ proteins ZO-1 and occludin via mechanisms involving MLCK and MLC phosphorylation.

## Introduction

Atherosclerosis (AS) is a multifactorial process associated with inflammation, which occurs in response to progressive vascular injury (1–3). Vascular endothelium has an important role in the atherosclerotic process, through the secretion of factors that can directly regulate vascular tone, induce accumulation and activation of platelets and leukocytes at the vessel wall, and cause proliferation of vascular smooth muscle cells (4–9). The endothelium forms barriers that define tissue compartments within higher organisms. Tight junctions (TJ) are essential to the functioning of the endothelial barrier (10–12). Endothelial TJs form a barrier against passive paracellular flux, which is regulated by complex physiological and pathophysiological signals that acutely control TJ permeability. The permeability of endothelial junctions is maintained by junction proteins that cross-link to the cytoskeleton. TJ proteins consist of occludin, claudins, junction adhesion molecules and zonulae occludentes (ZO). Occludin is positioned at cell junctions in close proximity to ZO-1, where they have a key role in maintaining the integrity of TJs and cell permeability (13,14).

Vascular endothelial dysfunction (VED) is a systemic disorder, and a critical element in the pathogenesis of AS and its complications. VED has recently gained attention as a key issue in cardiovascular biology, in particular with regard to its role in the origin and pathogenesis of AS, myocardial stunning, and restenosis following coronary artery balloon angioplasty (15). Previous studies have demonstrated that VED reflects a vascular phenotype prone to atherogenesis and may serve as a marker of inherent AS risk (16–18). Therefore, reversal of VED has been suggested as a novel therapeutic approach to inhibit or delay the incidence of AS.

Previous studies have implicated myosin light chain kinase (MLCK) in the regulation of endothelial cell (EC) barrier permeability through direct phosphorylation of myosin light chain (MLC) (19–22). MLCK is able to phosphorylate threo-nine 18 and serine 19 of MLC, which results in activation of intracellular cytoskeletal contraction-relaxation cycles (23). Phosphorylation induces a conformational change in MLC that enables actin-myosin interaction and cell contraction. Eutamene *et al* (24) used MLCK knockout mice to demonstrate that inhibition of MLCK activity can protect against acute lung injury.

Previous studies have demonstrated that endothelial permeability is increased in high-fat diet-induced AS (3,25); however, the precise underlying mechanisms have remained to be elucidated. The present study aimed to investigate whether the MLCK inhibitor ML7 is able to improve VED and AS by regulating the expression of TJ proteins ZO-1 and occludin via mechanisms involving MLCK and MLC phosphorylation in high-fat diet-fed rabbits.

## Materials and methods

### Ethics statement

All of the animal experimental and surgical procedures conducted in the present study were approved by the Animal Ethics Committee of the First Hospital Affiliated to Anhui Medical University (Hefei, China), in accordance with the National Guidelines for animal welfare (21).

### Reagents and instruments

Anti-MLCK monoclonal antibody (cat. no. M7905) and anti-phosphorylated MLC polyclonal antibody (cat. no. M6068) were purchased from Sigma-Aldrich (St. Louis, MO, USA), monoclonal antibodies targeting occludin (cat. no. ab167161), ZO-1 (cat. no. ab61357) and β-actin (cat. no. ab8226) were obtained from Abcam (Cambridge, UK). ML7, Oil red O (ORO) powder and acetylcholine (Ach) were purchased from Sigma-Aldrich. Nitroglycerin (NTG) was from Beijing Sihuan Pharmaceutical Co., Ltd. (Beijing, China). The total cholesterol (TC), low-density lipoprotein cholesterol (LDL-c), high-density lipoprotein cholesterol (HDL-c) and triglyceride (TG) ELISA kits were purchased from Beijing BHKT Clinical Reagent Co., Ltd. (Beijing, China). 3,3′-Diaminobenzidine (DAB) was obtained from Pierce Biotechnology, Inc. (Rockford, IL, USA). Polyvinylidene fluoride (PVDF) membranes were from GE Healthcare Bio-Sciences (Little Chalfont, UK). Horseradish peroxidase-conjugated secondary antibodies (cat. no. SP-9000-D) were purchased from Zhongshan Jinqiao Biotechnology Co., Ltd. (Beijing, China). Enhanced Chemiluminescence (ECL) reagents were obtained from Engreen Biosystem (Beijing, China). Vectashield mounting medium was from Vector Laboratories, Inc. (Burlingame, CA, USA). The 13-MHz ultrasound probe (GES6 two-dimensional Color Doppler Ultrasound Diagnostic Apparatus) was purchased from GE Healthcare Bio-Sciences. The DX51 light microscope was from Olympus Corporation (Tokyo, Japan).

### Animal groups and pre-treatment of tissue samples

A total of 49 two-month-old male New Zealand white rabbits (weighing 1.98±0.22 kg) were obtained from Nanjing Jinling Rabbit Farm (Nanjing, China), and were randomly divided into three groups. The rabbits were housed individually in screen-bottomed plastic cages, and maintained in a temperature-controlled room (25°C) with a standard 12 h light/dark cycle. The control group (n=14) was fed a standard diet for 12 weeks. The AS group (n=16) was fed a high-fat diet (standard diet supplemented with 5% lard and 2% cholesterol; Dalian Bell Pharmaceutical Co., Ltd., Dalian, China) for 12 weeks. The ML7 group (n=19) received a high-fat diet supplemented with ML7 (1 mg/kg/day) for 12 weeks. After 12 weeks, following fasting overnight, the rabbits were anesthetized with 50 mg/kg ketamine hydrochloride (Jiangsu Hengrui Medicine Co., Ltd., Jiangsu, China). Blood was collected for cholesterol determinations, and the aortas were excised and removed. One part of the aorta was harvested for ORO staining, and another was fixed in 4% formalin for immunohistochemistry (IHC) and hematoxylin & eosin (HE) staining. The aortas of the remaining rabbits were removed and immediately frozen at −80°C, prior to homogenization in 1X SDS lysis buffer (50 mM Tris-HCl, pH 6.8; 10% glycerol; 2% SDS) and western blot analysis.

### Transcutaneous non-invasive ultrasound measurement of vascular endothelial function

Transcutaneous non-invasive ultrasound evaluation of endothelial function of the abdominal aorta was performed two days prior to the end of the experiment, as described previously (26,27). The rabbits underwent a 12-h fast, and the marginal ear veins were cannulated for drug infusion. The rabbits were anesthetized with 3% pentobarbital sodium (Beijing Propbs Biotechnology Co., Ltd., Beijing, China) via the marginal ear vein, placed in the dorsal decubitus position and their abdomens were shaved. After 15 min of rest in the supine position, ultrasonic examination of the abdominal aorta was performed using a 13-MHz ultrasound probe. The transducer was lubricated with ultrasound gel (Shandong Beno Pharmaceutical Biotechnology Co., Ltd., Shandong, China) and placed at the abdominal aorta with minimal pressure, 1.0 cm below the renal artery, in order to obtain a longitudinal axis view of the abdominal aorta. Image settings were optimized to achieve the best and clearest definition of the endothelial-blood interface. Once the imaging of the aorta was considered optimal, the rabbits subsequently received the following sequential drug perfusions via the marginal ear vein for 2 min at 10-min intervals: i) saline (1 ml/min); ii) Ach at 1.5, 3 and 6 µg/ml/min; and iii) NTG at 5 and 7.5 µg/ml/min. Images of the abdominal aorta were continuously captured throughout the entire procedure at the largest cross section, and the maximal diameter was measured. The ultrasound probe remained in the same position for the duration of the measurement. The change in diameter was expressed as a percentage of the baseline diameter, denoted as Ratio 1, Ratio 2, Ratio 3, Ratio 4 and Ratio 5. Ratio refers to the aortic diastolic maximum inner diameter, under various doses of each drug vs. baseline aortic diastolic maximum inner diameter, under basal conditions. Ratios 1, 2 and 3 refer to aortic diastolic maximum inner diameter under Ach (1.5, 3 and 6 µg/ml/min) vs. the aortic diastolic maximum inner diameter under saline. Ratios 4 and 5 refer to aortic diastolic maximum inner diameter under NTG (5 and 7.5µg/ml/min) vs. the aortic diastolic maximum inner diameter under saline.

### Serum lipid measurement

Blood samples were collected via cardiac puncture, and serum TC, LDL-c, HDL-c, and TG levels were measured using commercially available ELISA kits. All measurements were performed according to the manufacturer's instructions and each sample was assayed in triplicate.

### ORO staining

Lipid content in the aorta wall was assessed using ORO staining. The ORO solution was prepared by slowly dissolving 0.5 g ORO powder in 100 ml isopropanol while heating to 60°C and stirring until completely dissolved. The solution was then filtered twice using Whatman filter paper (GE Healthcare Bio-Sciences) and cooled prior to use. The aortas were immersed in ORO solution for 20 min, followed by 1 min in 70% ethanol. Subsequently, the aortas were rinsed with 60% ethanol for 2 min followed by distilled water for several minutes. Images of the staining were then captured (D750; Nikon Corporation, Tokyo, Japan).

### HE staining

HE staining (Beijing Century Heli Biotechnology Co., Ltd., Beijing, China) was performed to evaluate the pathological and morphological changes in the arterial wall tissue. Fixed aortic specimens were dehydrated, embedded in paraffin, sectioned (6 µm) and stained with HE according to previously published methods (28). The tissue sections were deparaffinized using xylene, hydrated through a series of graded ethanol and stained with HE. The sections were subsequently dehydrated through a series of graded ethanol, cleared in xylene and mounted using neutral resin. The pathological and morphological changes of the arterial wall tissue were observed under an optical microscope (DM4000B, Leica Microsystems, Wetzlar, Germany).

### IHC analysis

The aortas were fixed in buffered parafor-maldehyde at 4°C and embedded in paraffin. The sections (6 µm) were deparaffinized, rehydrated and incubated in phosphate-buffered saline (PBS) containing 3% H_2_O_2_ in order to suppress endogenous peroxidase activity. The sections were then blocked in species-specific normal sera (Zhongshan Jinqiao Biotechnology Co., Ltd.) for 30–60 min in order to reduce non-specific staining and were subsequently incubated with primary antibodies (anti-MLCK, anti-phosphorylated MLC, anti-occludin, anti-ZO-1; 1:1,000) or pre-immune sera at 4°C overnight. The sections were then incubated with a horseradish peroxidase-conjugated secondary antibody. A DAB substrate working solution was used until the desired staining intensity was achieved to visualize the antibodies. Nuclei were counterstained with hematoxylin. Following thorough washing with PBS, the sections were mounted on glass slides using Vectashield and images were captured (Leica DM4000B).

### Western blot analysis

The aortas were washed three times in PBS and then lysed in 1X SDS lysis buffer containing 50 mM Tris-HCl (pH 6.8), 10% glycerol and 2% SDS (Sigma-Aldrich). The cell lysates were boiled for 10 min and then centrifuged at 16,060 x g for 15 min at room temperature. The protein samples (50 µg protein/lane) were then separated by 10% SDS-PAGE and transferred to PVDF membranes. The membranes were blocked in 5% bovine serum albumin (Sigma-Aldrich) for 2 h, followed by incubation at 4°C overnight with the appropriate primary antibodies (anti-occludin, 1:1,000; anti-ZO1, 1:1,000; anti-β-actin, 1:5,000). Primary antibodies were then detected following incubation with the corresponding horseradish peroxidase-conjugated secondary antibodies. Blots were visualized using ECL reagents and Kodak film (Eastman Kodak, Rochester, NY, USA). The results were analyzed using Image-Pro Plus 6.0 analysis system (Media Cybernetics, Inc., Rockville, MD, USA). To separate the membrane and cytoplasmic protein, a cell membrane and cytoplasm protein extraction kit (Beyotime Institute of Biotechnology, Shanghai, China) was used. Briefly, moderately homogenized cells were disrupted by low-speed centrifugation (700 x g, 10 min, 4°C) to remove nuclei and small amounts of precipitation produced by the cells. Subsequently, the cells underwent high-speed centrifugation (14,000 x g, 30 min, 4°C) in order to obtain the cell precipitate. The precipitate contained the membrane proteins, whereas the supernatant contained the cytoplasmic proteins. Membrane protein extraction agents were added to the precipitate and vortexed violently for 5 sec, in order to resuspend the precipitate, and the sample was incubated on ice for 5 min. The above steps were repeated twice and then centrifuged at 14,000 x g for 5 min at 4°C. The supernatant was then collected, which contained the membrane proteins.

### Statistical analysis

Values are expressed as the mean ± standard deviation. Student's t-test was used to identify significant differences between values. The correlation between vascular endothelial function and changes in serum lipid level was evaluated by Pearson correlation analysis. All statistical analyses were conducted using SPSS version 13.0 (SPSS Inc., Chicago, IL, USA). P<0.05 was considered to indicate a statistically significant difference.

## Results

### ML7 reduces lipid deposition lesions in AS rabbits

**Figure 1:**
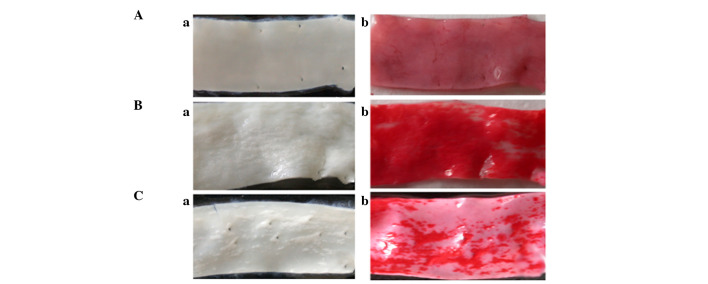
Treatment with ML7 reduces lipid deposition lesions in AS rabbits. A comparison was made between the appearance of arterial walls from rabbits in the control, AS and ML7 groups. Naked eye view of the arterial wall of (Aa) normal control group, (Ba) AS group and (Ca) ML7 group rabbits. Oil red O staining of the arterial wall from (Ab) control group, (Bb) AS group and (Cb) ML7 group rabbits. Atheromatous plaques were obvious in the AS group, and the number and area of the atheromatous plaques was reduced following ML7 treatment. AS, atherosclerosis.

The body weight of the rabbits increased in the AS and ML7 groups, as compared with that in the control group (P<0.01; Table I). In addition, the rabbits in the AS and ML7 groups exhibited arterial lesions (Fig. 1Ba and Ca) after being fed an atherogenic diet for 12 weeks, as compared with the control group (Fig. 1Aa). Lipid deposition in the lesions was detected using ORO staining (Fig. 1Ab–Cb). A reduction in the lesion area was observed in the rabbits of the ML7 group (Fig. 1Ca and Cb), as compared with the AS group (Fig. 1Ba and Bb). Administration of a high-fat diet significantly increased the serum lipid levels in the rabbits in the AS group, as compared with those in the rabbits in the control group. As shown in Table II, the serum levels of TC, LDL-c, HDL-c and TG were all markedly increased in the AS and ML7 groups as compared with those in the control group (P<0.01). Furthermore, the serum levels of TC and LDL-c were reduced, whereas levels of HDL-c were significantly increased in the ML7 group, as compared with those in the AS group (P<0.01).

### ML7 improves the vascular endothelial function of experimental AS

**Figure 2:**
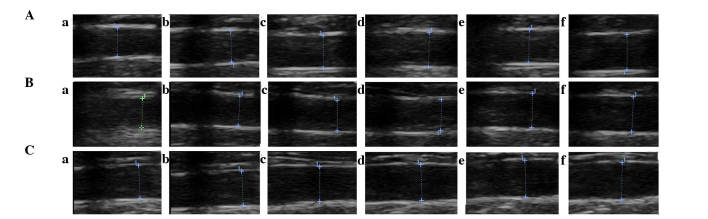
Treatment with ML7 improves the vascular endothelial function of experimental AS in rabbits. Vascular endothelial function was measured by transcutaneous noninvasive ultrasound. Longitudinal axis view of the abdominal aorta in B-mode ultrasound image. (A) Control group; (B) AS group; (C) ML7 group. Rabbits under sedation were serially infused for 2 min at 10-min intervals each in the marginal ear vein with (a) saline at 1 µg/ml/min, (b) Ach at 1.5 µg/ml/min, (c) Ach at 3 µg/ml/min, (d) Ach at 6 µg/ml/min, (e) NTG at 5 µg/ml/min and (f) NTG at 7.5 µg/ml/min. AS, atherosclerosis, Ach, acetylcholine; NTG, nitroglycerin.

The vascular endothelial function of the rabbits was compared between the three groups. Intake of the high-fat diet for 12 weeks did not produce significant alterations in endothelium-independent vasorelaxation of the abdominal aorta following NTG infusion; however, marked decreases were observed in endothelium-dependent vasorelaxation of the abdominal aorta following Ach infusion as compared with that in the control group (P<0.05 and P<0.01; Table III). Furthermore, treatment with ML7 significantly attenuated the high-fat diet-induced impairment of endothelium-dependent vasorelaxation of the abdominal aorta following Ach infusion (P<0.05 and P<0.01; Table III). Representative ultrasound images are shown in Fig. 2. These results indicated that ML7 markedly improved the vascular endothelial function of experimental AS in rabbits; however, it was unable to restore vascular endothelial function to its normal level.

In addition, a Pearson correlation analysis was conducted to evaluate the correlation between vascular endothelial function and the levels of serum lipids in the AS and ML7 groups. Pearson correlation analyses showed that alterations in vascular endothelial function were negatively correlated with changes in TC and LDL-c levels (P<0.01 and 0.05) and had little correlation with changes in TG and HDL-c levels in the AS and ML7 groups (Tables IV and V). These results indicated that treatment with ML7 improved vascular endothelial function by reducing the serum lipid levels.

### ML7 suppresses tight junction protein expression in AS rabbits

**Figure 3:**
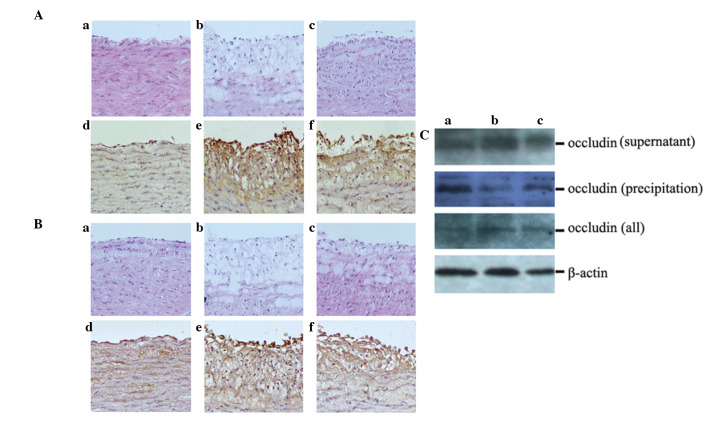
Treatment with ML7 blocks the expression of tight junction proteins ZO-1 and occludin in AS rabbits. (A) Expression of ZO-1 in the various rabbit groups. (a–c) HE stain; (d–f) IHC. (B) The expression of occludin in the various rabbit groups. (a–c) HE stain; (d–f) IHC. (C) Protein expression levels of occludin were analyzed by western blot analysis. (Aa, Ad, Ba, Bd and Ca) Control group; (Ab, Ae, Bb, Be and Cb) AS group; (Ac, Af, Bc, Bf and Cc) ML7 group. Magnification, x200. AS, atherosclerosis; ZO-1, zona occludens-1; IHC, immunohistochemistry; HE, hematoxylin and eosin.

A previous study by our group demonstrated that endothelial permeability is increased in high-fat diet-fed AS rabbits (29). Therefore, the present study assessed the changes in endothelial TJ proteins in response to ML7 by using HE and IHC staining as well as western blot analysis. HE staining showed that, compared with the control group (Fig. 3Aa and Ba), hyperplasia was evident in the uneven aortic intima, and the ECs had an incomplete structure in the AS group (Fig. 3Ab and Bb). Treatment with ML7 smoothed the intima, and fewer foam cells, vascular smooth muscle cells and lipid plaques were observed underneath the endothelium (Fig. 3Ac and Bc). For IHC, the sections were incubated with antibodies targeting the TJ proteins ZO-1 and occludin. The results demonstrated that ZO-1 and occludin were not as highly expressed in the control group (Fig. 3Ad and Bd) as in the AS group (Fig. 3Ae and Be), whereas treatment with ML7 reduced the expression levels of ZO-1 and occludin (Fig. 3Af and Bf). Western blot analysis produced similar results regarding occludin expression in the total protein lysates of rabbit aortas. It was further demonstrated that treatment with ML7 increased occludin expression in the precipitate, but reduced its expression in the supernatant of lysed aortas, thus indicating that occludin expression occurred during remodeling from cell membrane to cytoplasm in AS (Fig. 3C).

### ML7 attenuates MLCK expression and MLC phosphorylation in AS rabbits

**Figure 4:**
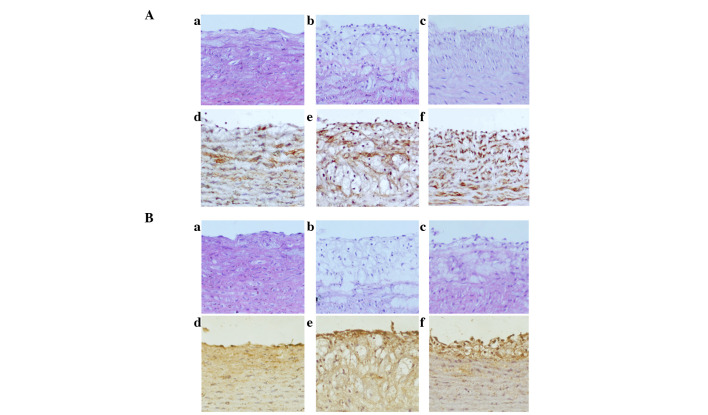
Expression of MLCK and phosphorylation of MLC was increased in AS rabbits, whereas treatment with ML7 attenuated MLCK expression and MLC phosphorylation. (A) Expression of MLCK in the various rabbit groups. (a–c) HE stain; (d–f) IHC. (B) Phosphorylation of MLC in the various rabbit groups. (a–c) HE stain; (d–f) IHC. (Aa, Ad, Ba and Bd) Control group; (Ab, Ae, Bb and Be) AS group; (Ac, Af, Bc and Bf) ML7 group. Magnification, x200. AS, atherosclerosis; MLC, myosin light chain; MLCK, MLC kinase; IHC, immunohistochemistry; HE, hematoxylin and eosin.

To further elucidate the precise mechanisms underlying ML7 function, MLCK expression and MLC phosphorylation were examined using HE staining and IHC. HE staining of MLCK (Fig. 4Aa–c) and phosphorylated MLC (Fig. 4Ba–c) showed that the arterial specimens of the control group exhibited a normal morphology of the aorta intima on the luminal side (Fig. 4Aa and Ba). In the AS group, the ECs were shown to have an incomplete structure (Fig. 4Ab and Bb), whereas treatment with ML7 smoothed the intima (Fig. 3Ac and Bc). IHC staining of the sections with anti-MLCK and anti-phosphorlyated MLC antibodies showed that the expression of MLCK and the phosphorylation of MLC was markedly increased in AS rabbits (Fig. 4Ae and Be) as compared with that in the control group (Fig. 4Ad and Bd). Positive staining was distributed regularly, and was most intense in areas surrounding the luminal side. Treatment with ML7 attenuated MLCK expression and MLC phosphorylation in the aortic ECs (Fig. 4Af and Bf).

## Discussion

Vascular ECs are the primary permeability barrier between vascular tissue and blood, and are important for the maintenance of normal biological homeostasis (30). It is generally hypothesized that AS lesions are initiated by arterial endothelial injury. A previous study by our group showed that endothelial permeability was increased in high-fat diet-fed AS rabbits (29). MLCK is a protein kinase that has an important role in the re-organization of the cytoskeleton, leading to disruption of vascular barrier integrity (31). In the present study, higher serum TC, LDL-c and TG levels, and typical morphological characteristics of arterial lesions were observed in the rabbits of the AS group as compared with those in rabbits fed a normal diet. Treatment with the MLCK inhibitor ML7 markedly reduced serum levels of TC, LDL-c and TG, and increased HDL-c levels, as well as inhibiting the progression of AS in high-fat diet-fed rabbits. MLCK catalyzes the phosphorylation of MLC, leading to cytoskeletal re-arrangements. EC concentric contraction and gap formation are followed by alterations to the cytoskeleton, causing lipid infiltration into the arterial intima, which accumulate in arterial walls, ultimately leading to atheromatous plaque formation (32–34). Numerous studies have indicated that hyperlipidemia is an important risk factor for the occurrence of AS (2,35).

A study on the basic mechanisms associated with atherogenesis indicated that VED represents a key early step in the development of AS, and is also associated with plaque progression and the occurrence of atherosclerotic complications (17). In the present study, transcutaneous non-invasive ultrasound evaluation of endothelial function in AS rabbits demonstrated a marked decrease in endothelium-dependent vasorelaxation of the abdominal aorta following Ach infusion as compared with that in the rabbits fed a normal diet. Treatment with ML7 attenuated the high-fat diet-induced impairment of endothelium-dependent vasorelaxation and improved the VED by reducing serum lipid levels. These findings suggested important roles for ML7 in the amelioration of lipid metabolism, VED and AS in high-fat diet-fed rabbits.

The present study also demonstrated that MLCK and MLC phosphorylation were clearly involved in TJ regulation. The permeability of endothelial junctions is maintained by TJ proteins, including ZO-1 and occludin, which cross-link to the cytoskeleton (36). The results of the present study demonstrated that ZO-1 and occludin were highly expressed in the AS group, whereas treatment with ML7 was able to reduce the expression levels of ZO-1 and occludin. Western blot analysis showed similar results regarding occludin expression in the total protein lysates of the rabbit aortas. Furthermore, it was demonstrated that treatment with ML7 increased occludin expression in the precipitate, but reduced its expression in the supernatant of lysed aortas, thus indicating that occludin expression occurred during remodeling from cell membrane to cytoplasm.

ML7 is a selective MLCK inhibitor, which acts on the adenosine triphosphate-binding site of the active center of MLCK (21). The results of the present study confirmed the crucial role of MLCK in vascular endothelial permeability regulation. Although the number of studies on actomyosin regulation of cell morphology has increased over the past few years, the functional importance of the contractile elements in controlling permeability remains to be elucidated (37). The present study demonstrated that MLCK expression and MLC phosphorylation were significantly increased in AS rabbits, and treatment with ML7 attenuated MLCK expression and MLC phosphorylation in aortic ECs, thus indicating that TJ protein expression, endothelial permeability and gap formation may be associated with MLCK expression and MLC phosphorylation. The present study was the first, to the best of our knowledge, to indicate that MLCK inhibitor ML7 may improve VED and AS by regulating the expression of TJ proteins ZO-1 and occludin via mechanisms involving MLCK and MLC phosphorylation in high-fat diet-fed rabbits.

In conclusion, the present study provided evidence that ML7 is able to regulate lipid metabolism, and improve VED and AS in high-fat diet-fed rabbits. MLCK-mediated MLC phosphorylation was shown to have an important role in the development of AS. However, further studies are required to define the mechanism underlying this regulation and clarify the associated signaling networks.

## Figures and Tables

**Table I:** Effects of a high-fat diet on the weight of experimental rabbits.

Group	0-week body weight (kg)	12-week body weight (kg)
Control (n=14)	1.92±0.18	2.50±0.22
AS (n=16)	2.00±0.24	2.96±0.32^a^
ML-7 (n=19)	2.02±0.24	2.82±0.29^a^

Values are expressed as the mean ± standard deviation.

a P<0.01 vs. control group. AS, atherosclerosis.

**Table II:** Serum lipid levels in the various groups.

Lipid (mmol/l)	Control group (n=14)	AS group (n=16)	ML-7 group (n=19)
TC	1.52±0.51	29.37±4.36^a^	20.60±4.43^a^,^b^
LDL-c	0.73±0.31	24.57±4.57^a^	17.27±3.93^a^,^b^
HDL-c	0.64±0.34	2.05±1.39^a^	4.34±0.68^a^
TG	0.23±0.10	2.72±2.42^a^	1.62±0.81^a^

Values are expressed as the mean ± standard deviation.

a P<0.01 vs. control group;

b P<0.01 vs. AS group. AS, atherosclerosis; TC, total cholesterol; LDL-c low-density lipoprotein cholesterol; HDL-c, high-density lipoprotein cholesterol; TG, triglycerides.

**Table III:** Changes in the abdominal aorta diameter in response to acetylcholine or nitroglycerin infusions in the three groups.

Ratio	Control group	AS group	ML-7 group
1	8.84±3.81	2.04±2.07^a^	5.40±3.05^b^,^d^
2	13.56±2.86	4.32±2.38^a^	7.79±3.60^a^,^d^
3	16.96±3.67	5.35±1.96^a^	10.95±3.836^a^,^c^
4	14.08±2.04	14.56±4.23	15.36±3.88
5	18.55±4.05	17.92±4.70	18.29±3.52

Values are presented as a percentage of the baseline diameter, and are expressed as the mean ± standard deviation.

a P<0.01,

b P<0.05 vs. control group;

c P<0.01,

d P<0.05 vs. AS group. AS, atherosclerosis.

**Table IV:** Pearson correlation analysis between vascular endothelial function and the levels of serum lipids in the atherosclerosis group.

Ratio	TC	LDL-c	HDL-c	TG
1	−0.821^a^	−0.850^a^	0.115	0.085
2	−0.565	−0.668^b^	0.104	0.313
3	−0.891^a^	−0.759^b^	−0.048	−0.261

a P<0.01,

b P<0.05. TC, total cholesterol; LDL-c, low-density lipoprotein cholesterol; HDL-c, high-density lipoprotein cholesterol; TG, triglycerides.

**Table V:** Pearson correlation analysis between vascular endothelial function and the levels of serum lipids in the ML-7 group.

Ratio	TC	LDL-c	HDL-c	TG
1	−0.798^b^	−0.867^a^	0.290	−0.531
2	−0.807^a^	−0.804^a^	0.154	−0.751^b^
3	−0.795^b^	−0.756^b^	−0.016	−0.814^a^

a P<0.01,

b P<0.05. TC, total cholesterol; LDL-c, low-density lipoprotein cholesterol; HDL-c, high-density lipoprotein cholesterol; TG, triglycerides.
